# Pharmacogenomic Variants May Influence the Urinary Excretion of Novel Kidney Injury Biomarkers in Patients Receiving Cisplatin

**DOI:** 10.3390/ijms18071333

**Published:** 2017-06-22

**Authors:** Cara Chang, Yichun Hu, Susan L. Hogan, Nickie Mercke, Madeleine Gomez, Cindy O’Bryant, Daniel W. Bowles, Blessy George, Xia Wen, Lauren M. Aleksunes, Melanie S. Joy

**Affiliations:** 1 Department of Pharmaceutical Sciences, Skaggs School of Pharmacy and Pharmaceutical Sciences, University of Colorado, Aurora, CO 80045, USA; Cara.Chang@ucdenver.edu (C.C.); Nickie.Mercke@ucdenver.edu (N.M.); Marie.Gomez@ucdenver.edu (M.G.); Cindy.O’Bryant@ucdenver.edu (C.O.); 2 Kidney Center, University of North Carolina School of Medicine, Division of Nephrology and Hypertension, Chapel Hill, NC 27599, USA; Yichun_Hu@med.unc.edu (Y.H.); Susan_Hogan@ucdenver.edu (S.L.H.); 3 Cancer Center, University of Colorado, Aurora, CO 80045, USA; Daniel.Bowles@ucdenver.edu; 4 Department of Pharmacology and Toxicology, Ernest Mario School of Pharmacy, Rutgers University, Piscataway, NJ 08854, USA; Blessyg@scarletmail.rutgers.edu (B.G.); wen@eohsi.rutgers.edu (X.W.); aleksunes@eohsi.rutgers.edu (L.M.A.); 5 Division of Renal Diseases and Hypertension, University of Colorado School of Medicine, Aurora, CO 80045, USA

**Keywords:** cisplatin, nephrotoxicity, acute kidney injury, pharmacogenomics, CTR1, GGT1, GST, KEAP1, MATE1, MRP2, NRF2, OCT2

## Abstract

Nephrotoxicity is a dose limiting side effect associated with the use of cisplatin in the treatment of solid tumors. The degree of nephrotoxicity is dictated by the selective accumulation of cisplatin in renal tubule cells due to: (1) uptake by organic cation transporter 2 (OCT2) and copper transporter 1 (CTR1); (2) metabolism by glutathione S-transferases (GSTs) and γ-glutamyltransferase 1 (GGT1); and (3) efflux by multidrug resistance-associated protein 2 (MRP2) and multidrug and toxin extrusion protein 1 (MATE1). The purpose of this study was to determine the significance of single nucleotide polymorphisms that regulate the expression and function of transporters and metabolism genes implicated in development of acute kidney injury (AKI) in cisplatin treated patients. Changes in the kidney function were assessed using novel urinary protein biomarkers and traditional markers. Genotyping was conducted by the QuantStudio 12K Flex Real-Time PCR System using a custom open array chip with metabolism, transport, and transcription factor polymorphisms of interest to cisplatin disposition and toxicity. Traditional and novel biomarker assays for kidney toxicity were assessed for differences according to genotype by ANOVA. Allele and genotype frequencies were determined based on Caucasian population frequencies. The polymorphisms rs596881 (*SLC22A2*/OCT2), and rs12686377 and rs7851395 (*SLC31A1*/CTR1) were associated with renoprotection and maintenance of estimated glomerular filtration rate (eGFR). Polymorphisms in *SLC22A2*/OCT2, *SLC31A1*/CTRI, *SLC47A1*/MATE1, *ABCC2*/MRP2, and *GSTP1* were significantly associated with increases in the urinary excretion of novel AKI biomarkers: KIM-1, TFF3, MCP1, NGAL, clusterin, cystatin C, and calbindin. Knowledge concerning which genotypes in drug transporters are associated with cisplatin-induced nephrotoxicity may help to identify at-risk patients and initiate strategies, such as using lower or fractionated cisplatin doses or avoiding cisplatin altogether, in order to prevent AKI.

## 1. Introduction

While effective as a chemotherapeutic agent for various solid tumors [1,2,3,4,5,6,7], cisplatin has been limited by its toxicities. Cisplatin treatment can result in nephrotoxicity, ototoxicity, neurotoxicity, infections, and gastrointestinal toxicity secondary to its high emetogenic potential. Of these adverse events, acute kidney injury (AKI) is a prominent toxicity. Approximately one-third of patients treated with a single dose of cisplatin (50–100 mg/m^2^) will experience kidney impairment [8,9]. It is known that cisplatin selectively accumulates in the proximal tubule, leading to oxidative stress, inflammation, and vascular injury, and subsequent renal pathology [10]. The degree to which cisplatin and its metabolites accumulate and induce injury can be influenced by the coordinated activity of uptake and efflux transport proteins on the basolateral and apical membranes of the kidney proximal tubules [11]. Cisplatin metabolism is also important in governing proximal tubule exposure to the parent drug and its conjugates [12].

Single nucleotide polymorphisms in cisplatin metabolism and transport genes are hypothesized to modulate risk of nephrotoxicity. Uptake of cisplatin by kidney proximal tubule cells is governed by organic cation transporter 2 (OCT2) [13] and copper transporter 1 (CTR1) [14] on the basolateral membrane. Studies using mice lacking the Oct1 and -2 transporters have observed reduced kidney exposure to cisplatin and attenuation of nephrotoxicity [13,15]. In the proximal tubule cell, cisplatin is metabolized by glutathione *S*-transferase pi-1 (GSTP1) and γ-glutamyltransferase 1 (GGT1) enzymes. The function of GST and GGT1 enzymes can also contribute to cisplatin induced nephrotoxicity [12]. Metabolism of cisplatin by GST results in a cisplatin-glutathione substrate that undergoes efflux from the proximal tubule cell and is subsequently metabolized extracellularly at the apical brush border membrane by GGT1 [12]. The resultant substrate, a cisplatin-cysteinyl-glycine conjugate, is metabolized to cisplatin-cysteine by aminodipeptidase and reabsorbed into the proximal tubule cell [12]. Subsequent metabolism processes in the proximal tubule cell lead to the development of a reactive thiol that enhances nephrotoxicity [12]. This mechanism is supported by studies in mice lacking GGT, showing protection against cisplatin induced kidney injury [16]. Cisplatin metabolites are extruded from the proximal tubule cell into urine via brush border (apical) efflux transporters including multidrug and toxin extrusion protein 1 (MATE1) and by multidrug resistance-associated protein 2 (MRP2) [17]. MATE1- and MRP2-null mice display increased cisplatin induced kidney toxicity and experience increased renal exposure to cisplatin [18,19]. Although knockout studies in animals have supported genetic influences on cisplatin nephrotoxicity, there are limited studies to date that have evaluated the role of genetics of metabolism enzymes and transporters on risk of cisplatin nephrotoxicity in humans [20].

Regulatory proteins, such as Kelch-like ECH-associated protein 1 (KEAP1) and nuclear factor (erythroid-derived 2)-like 2 (NRF2), can modulate the expression of metabolism and transport processes including GSTs and MRP2 [21,22,23]. KEAP1 acts as a regulatory protein that represses the activity of NRF2 and promotes its degradation by ubiquitination [24]. NRF2 is a transcription factor that binds to antioxidant response elements (AREs) in the regulatory regions of target genes that direct drug metabolism, transport, detoxification, and antioxidant function [21,22,23]. Mice lacking NRF2 are reported to experience enhanced cisplatin nephrotoxicity, suggesting a role for NRF2 in protecting the kidney [25].

Serum creatinine has been accepted as the clinical biomarker for determination of kidney function and toxicity following cisplatin exposure. However, other novel protein biomarkers may be more sensitive and specific markers of AKI [26]. Based on the current Kidney Disease Improving Global Outcomes (KDIGO) Guidelines, AKI is defined by a rapid decline in kidney function based on an increase of serum creatinine (≥0.3 mg/dL within 48 hours or ≥1.5 times baseline within the previous seven days) and a decrease in urine output (<0.5 mL/kg/h for six hours) [27,28]. Compared to traditional clinical markers, kidney injury molecule-1 (KIM-1), calbindin, trefoil factor 3 (TFF3), and cystatin C are urinary biomarker candidates with enhanced sensitivity for assessing kidney injury due to drugs such as cisplatin [29,30,31,32]. Relationships between urinary biomarkers, as indicators of cisplatin kidney injury, and polymorphisms in candidate drug metabolism and transport genes have not previously been evaluated.

The current study sought to elucidate the influence of polymorphisms in drug transport, metabolism, and regulatory genes on cisplatin-induced nephrotoxicity by assessing changes in novel urinary protein biomarkers as subclinical measures of injury.

## 2. Results

### 2.1. Patient Characteristics

A total of 206 patients who were scheduled to receive or who had received cisplatin were recruited. This included prospective (*n* = 57) and retrospective (*n* = 149) patients. For the prospective patients, a subset (*n* = 28) were recruited prior to the first cycle of cisplatin-containing chemotherapy, while another subset (*n* = 29) was recruited prior to the second cycle of cisplatin. The average time between first and second dose was 17 days (range of 6–34 days). Prospective patients contributed both genetics and urinary biomarker data, while retrospective patients contributed only genetics data. All patients were adequately hydrated and were not exposed to any concomitant nephrotoxins. Patient demographic and baseline laboratory characteristics are shown in Table 1 [33]. The commonly co-prescribed (greater than 10%) chemotherapeutic agents included etoposide (*n* = 38, 18%), vinblastine (*n* = 32, 16%), dacarbazine (*n* = 29, 14%), aldesleukin (*n* = 28, 14%), interferon alfa 2b (*n* = 27, 13%), gemcitabine (*n* = 23, 11%), and docetaxel (*n* = 23, 11%). The allelic frequencies were in Hardy-Weinberg Equilibrium (HWE) (Table 2). The expected frequencies of minor and major alleles used in the HWE determinations were for the Caucasian (EUR) population in HapMap, which reflected the majority (92%) of patients enrolled in the study.

### 2.2. Associations between SLC22A2 and SLC31A1 Variants and Estimated Glomerular Filtration Rate (eGFR) in Patients Receiving Cisplatin

**Figure 1:**
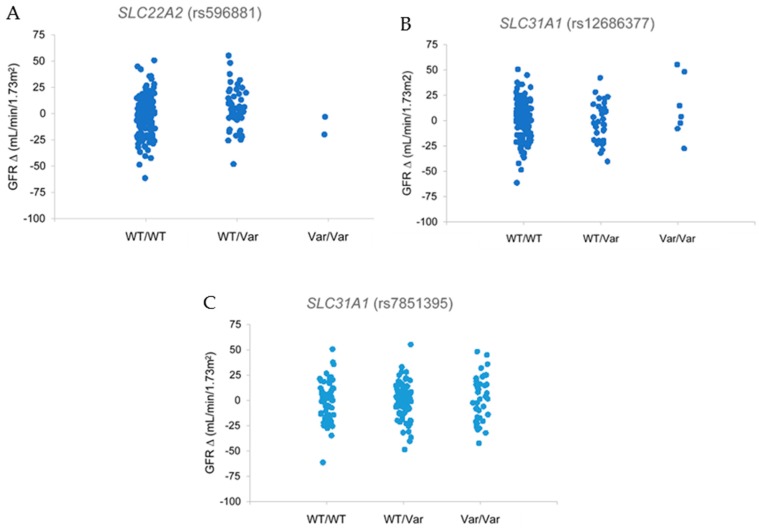
(**A**) Comparisons of *SLC22A2* (rs596881) genotypes on eGFR in ambulatory cancer patients prescribed cisplatin. Patients carrying a variant allele of rs596881 in *SLC22A2* exhibited statistically significant increases in eGFR (*p* = 0.01). (**B**,**C**) Comparisons of *SLC31A1* (rs12686377 and rs7851395) genotypes on eGFR in ambulatory cancer patients prescribed cisplatin. Homozygous variant patients for *SLC31A1* (rs12686377 and rs7851395) exhibited eGFR protection with cisplatin therapy (*p* = 0.01 and *p* = 0.04). Graphs indicate percent changes in eGFR from baseline. Abbreviations: Estimated glomerular filtration rate: eGFR, Wildtype: WT, Variant: VAR.

Relationships between clinical kidney injury and polymorphisms in transporter, metabolism, and regulatory genes were assessed using estimated glomerular filtration rate (eGFR), a standard clinical measure of kidney function and overt nephrotoxicity. Genetic variants in the two cisplatin uptake transporters *SLC22A2* (OCT2) and *SLC31A1* (CTR1) were associated with preservation of kidney function. Patients with the CT genotype in *SLC22A2* polymorphism rs596881 exhibited positive changes in eGFR compared to individuals with the wildtype CC genotype (Figure 1A, *p* = 0.01). Similarly, increases in eGFR were observed in patients with both *SLC31A1* variants (rs12686377 and rs7851395). Patients with the GG (rs12686377) and AA (rs7851395) variant genotypes had positive changes in eGFR (*p* = 0.01 and *p* = 0.04) compared to patients with the wildtype genotypes AA and CC, respectively (Figure 1B,C). Taken together, these data support renoprotective properties for some genetic variants in cisplatin uptake transporters. By comparison, genetic variants for the efflux transporters *SLC47A1* and *ABCC2* or metabolism genes *GGT1* and *GSTP1* were not associated with changes in eGFR.

Interestingly, patients with the CG genotype for a *KEAP1* variant (rs1048290) had an increase in eGFR compared to wildtype CC patients (*p* = 0.03). In addition, patients with the GG variant (rs2886162) in the redox sensor *NFE2L2* exhibited eGFR improvement relative to wildtype AA patients (*p* = 0.04). 

### 2.3. Associations between Transporter Gene Variants and Novel Urinary Biomarkers of Kidney Injury in Patients Receiving Cisplatin

**Figure 2:**
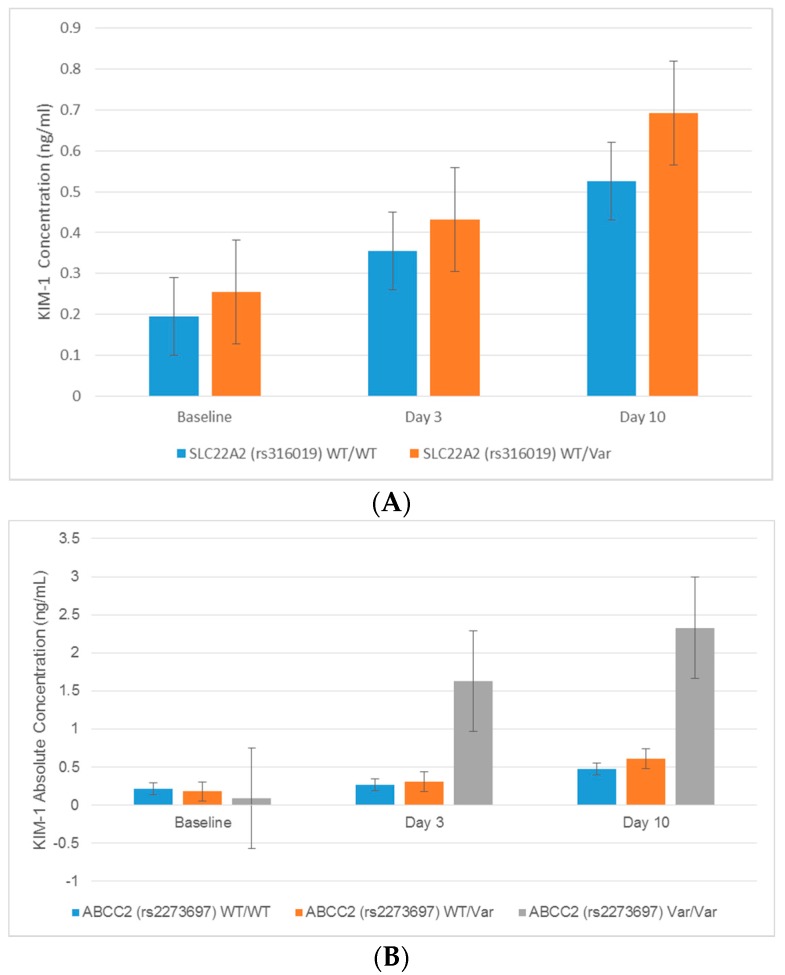
(**A**) Comparison of absolute KIM-1 concentrations by *SLC22A2* (rs316019) genotype in ambulatory cancer patients prescribed cisplatin. Statistically significant increases of KIM-1 at baseline (*p* = 0.02), Day 3 (*p* = 0.03), and Day 10 (*p* = 0.046) were demonstrated in patients expressing the SLC22A2 rs316019 variant. (**B**) Comparison of absolute KIM-1 concentrations to *ABCC2* (rs2273697) genotype in ambulatory cancer patients prescribed cisplatin. Statistically significant increases of KIM-1 at baseline (*p* = 0.02), Day 3 (*p* = 0.03) and Day 10 (*p* = 0.046) were demonstrated in patients expressing the *ABCC2* rs2273697 variant. Error bars represent standard deviations. Abbreviations: Kidney injury molecule 1: KIM-1, Wildtype: WT, Variant: Var.

Associations between transporter gene polymorphisms and novel urinary biomarkers generally suggested that variant alleles were predictive of increases in the urinary excretion of kidney injury biomarkers in patients receiving cisplatin, although there were a few notable exceptions (Table 3). Significant changes in urinary biomarker excretion were associated with the uptake transporter genes *SLC22A2* and *SLC31A1*. Patients with the AC genotype for the *SLC22A2* rs316019 polymorphism exhibited higher urinary fold changes in KIM-1 at baseline (*p* = 0.02), Day 3 (*p* = 0.03), and Day 10 (*p* = 0.046) compared to the wildtype CC genotype (Figure 2, Table 3). Patients with a CT genotype in the *SLC22A2* variant rs596881 were also associated with a significant decrease in β2 microglobulin (B2M) and increase in osteopontin at Day 3 after cisplatin relative to patients with the wildtype CC genotype (Table 3). It should be noted that patients expressing two copies of the variant alleles for the rs316019 and rs596881 variants in *SLC22A2* did not exhibit significant changes in urinary biomarkers due to their small numbers (*n* = 3 rs316019 and *n* = 1 rs596881). In addition to *SLC22A2*, a positive association between *SLC31A1* and a urinary biomarker was observed. Patients with the GG variant in rs7851395 had greater increases in urinary osteopontin levels at Day 3 after cisplatin treatment (Table 3). 

**Figure 3:**
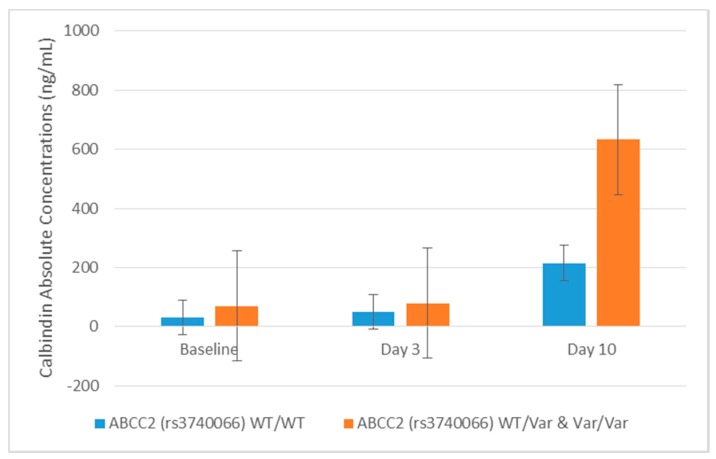
Comparison of calbindin absolute concentrations to *ABCC2* (rs3740066) genotype in ambulatory cancer patients prescribed cisplatin. Absolute concentrations of calbindin were elevated in patients with the variant allele at all time points; statistically significant at baseline (*p* = 0.047), Day 3 (*p* = 0.02), and Day 10 (*p* = 0.02) versus homozygous wildtype patients. Error bars represent standard deviations. Abbreviations: Wild type: WT, Variant: Var.

The AA genotype for the rs2289669 variant in the *SLC47A1* gene was associated with fold change elevations in KIM-1 (*p* = 0.007) and monocyte chemoattractant protein-1 (MCP-1, *p* = 0.015) at Day 3 after cisplatin administration compared to patients with the wildtype GG genotype. In addition, variants in the *ABCC2* gene were predominantly associated with higher concentrations of AKI-associated urinary biomarkers. The *ABCC2* variant rs3740066 was associated with enhanced calbindin levels in urine after cisplatin administration. Calbindin concentrations were significantly elevated by 2.2-, 1.9-, and 2.7-fold at baseline, Day 3, and Day 10, respectively, in variant (TT vs. CC) genotype patients (Table 3, Figure 3). The *ABCC2* rs3740066 polymorphism was also associated with statistically significant increases in the urinary excretion of clusterin, cystatin C, and NGAL at Day 3 (Table 3). In addition to the rs3740066 variant, patients with the CT genotype for the rs717620 *ABCC2* variant exhibited a 3.5-fold increase in clusterin and 2.6-fold enhancement of cystatin C concentrations at Day 3 compared to the wildtype CC genotype (Table 3). Few patients were homozygous for the rs3740066 and rs717620 variants in *ABCC2* and, in turn, no significant associations were observed with the excretion of urinary biomarkers following cisplatin treatment. 

### 2.4. Association between Cisplatin Metabolism Genes and Novel Urinary Biomarkers of Kidney Injury in Patients Receiving Cisplatin

Patients with the GG genotype for the *GSTP1* rs1695 variant had significant elevations in urinary biomarkers indicative of AKI compared to wildtype AA patients. They exhibited two-fold or greater increases in urinary KIM-1, calbindin, and NGAL concentrations at Day 3 and urinary IL-18 at Day 10 (Table 3).

### 2.5. Association between Regulatory Genes and Novel Urinary Biomarkers of Kidney Injury in Patients Receiving Cisplatin

A few of the evaluated *NFE2L2* variants were associated with significant fold changes in the excretion of urinary biomarkers. Homozygous variants of *NFE2L2* rs1806649, rs1962142, rs2886162, and rs2706110 were associated with statistically significant fold-change increases in IL-18, TFF3, calbindin, and MCP-1, respectively, versus homozygous wildtypes (Table 3). 

**Figure 4:**
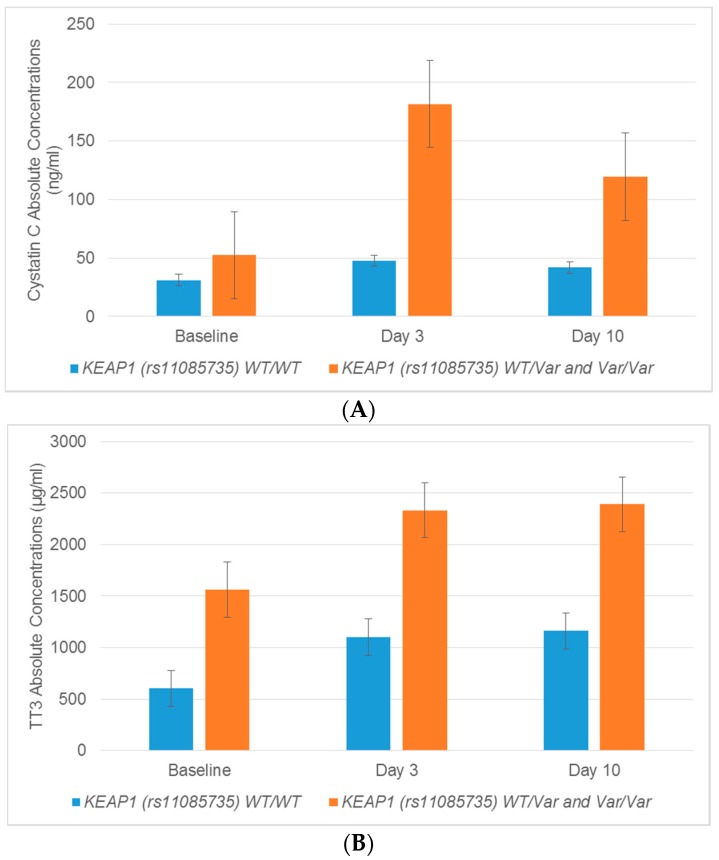
Variant alleles in *KEAP1* (rs11085735) are correlated with increased concentrations of TFF3 and cystatin C. (**A**) Absolute concentrations of cystatin C showed a statistically significant increase on Day 3 (*p* = 0.01) and Day 10 (*p* = 0.03) in patients expressing the variant allele of *KEAP1*. (**B**) Absolute concentrations of TFF3 were increased at baseline (*p* = 0.01), Day 3 (*p* = 0.03) and Day 10 (*p* = 0.03) in patients who carried at least one variant copy of *KEAP1*. Error bars represent standard deviations. Abbreviations: Trefoil factor: TFF3, Wildtype: WT, Variant: Var.

The *KEAP1* polymorphism rs11085735 was associated with the excretion of several urinary biomarkers in patients who were heterozygous versus homozygous wildtype. Cystatin C and TFF3 fold changes were statistically increased on Day 3 and Day 10 in patients with the AC variant genotype compared to wildtype CC patients (Table 3, Figure 4). Cystatin C concentrations on Day 3 and Day 10 were enhanced by 3.3- and 2.6-fold, respectively, in patients with at least one copy of the *KEAP1* rs11085735 variant (AC) compared to patients with the reference alleles (CC) (*p* = 0.011, *p* = 0.027) (Table 3). Elevations in TFF3 were 2.5-, 1.8-, and 1.8-fold in patients with the AC genotype for rs11085735 compared to wildtype CC individuals at baseline, Day 3, and Day 10, respectively (*p* = 0.006, *p* = 0.038, *p* = 0.034) (Table 3). There were limited numbers of patients with the AA genotype for the rs11085735 polymorphism in *KEAP1*; in turn, no significant associations were observed with urinary biomarkers.

## 3. Discussion

The current study sought to address the role of variants in drug transport, metabolism, and regulatory genes on cisplatin-induced nephrotoxicity in cancer patients by assessing changes in the urinary concentrations of novel protein biomarkers as markers of subclinical injury to the kidneys. The results demonstrate associations that support the role of transporter and metabolizing enzyme genetics on risk or mitigation of risk of kidney injury by cisplatin. While specific variants in the uptake transporters *SLC22A2* (rs596881) and *SLC31A1* (rs12686377 and rs7851395) were associated with preserved eGFR, respectively, other variants in these genes were associated with fold-changes in novel urinary biomarkers. Variants in the efflux transporter genes *ABCC2* and *SLC47A1* were associated with elevations in novel urinary biomarkers, but not eGFR. The *SLC47A1* variant rs2289669 correlated with enhanced KIM-1 and MCP-1 concentrations. Significantly higher levels of KIM-1 at Day 3 were observed in patients with the *ABCC2* variant rs2273697. The *ABCC2* rs3740066 variant was associated with consistently enhanced fold-changes in calbindin, cystatin C, clusterin, and NGAL in patients who carried one or two alleles. Patients who carried the variants in rs1695 (*GSTP1*) have higher urinary concentrations of several biomarkers including KIM-1, calbindin, NGAL, and IL-18. Lastly, since regulatory genes such as *NFE2L2* and *KEAP1* can indirectly influence the effects of transporter and drug metabolizing enzyme genes [34,35], associations between polymorphisms in these genes and novel urinary biomarkers were also evaluated. The *NFE2L2* polymorphism rs2886162 was consistently associated with enhanced calbindin excretion from baseline, while the *KEAP1* polymorphism rs11085735 was associated with increases in TFF3 and cystatin C concentrations. The current study is the first report to date in a cisplatin-treated patient cohort that has evaluated associations between targeted pharmacogenomics of drug transport and metabolism genes and a panel of novel urinary biomarkers of kidney injury. A follow-up validation study will be needed to confirm the findings from the current study.

The importance of the OCT2 uptake transporter in cisplatin renal clearance was previously reported in a study employing Oct1/2 mouse knockouts [20]. These authors subsequently evaluated changes to serum creatinine after treatment with cisplatin in human cancer patients (*n* = 78) according to the presence or absence of a copy of the nonsynonymous *SLC22A2* rs316019 variant denoting reduced OCT2 function [20]. The presence of the *SLC22A2* variant was associated with maintenance of serum creatinine. The *SLC22A2* polymorphism rs316019 (G808T; Ser270Ala) has also been associated with protection from cisplatin ototoxicity [36], odds of hepatotoxicity and hematologic toxicity secondary to platinum chemotherapy [37], and increased metformin renal and secretory clearance [38]. In the current study, patients heterozygous vs. homozygous wildtype for the *SLC22A2* rs316019 polymorphism had associated higher concentrations and fold-changes in urinary KIM-1 at baseline, Day 3, and Day 10 as compared to wildtype homozygotes. The *SLC22A2* rs596881, rs3127573, and rs2279463 variants were associated with elevations in B2M at Day 3. In addition, in the current study, patients with the *SLC22A2* rs596881 variant exhibited a preservation of eGFR. A prior study has associated the *SLC22A2* rs3127573 variant with increased OCT2 function [39]. Based on our data and the supporting literature, variants in the *SLC22A2* gene were related to risk or mitigation of risk for cisplatin induced kidney injury.

A previous study has reported on a genetic variant (rs10981694) in the copper transporter protein 1 (*SLC31A1*) and cisplatin-induced ototoxicity [40]. An additional study observed increased platinum resistance in lung cancer patients that was associated with two *SLC31A1* variants (rs7851395 and rs12686377) [41]. The current study reported significant fold increases in eGFR in homozygous variant versus homozygous wildtype patients (*p* = 0.01 and *p* = 0.04) with the previously reported variants (rs12686377 and rs7851395) in the *SLC31A1* gene. We also found higher osteopontin concentrations associated with homozygosity for the *SLC31A1* variant rs7851395 at Day 3 post cisplatin treatment. These studies support the role of *CTR1* variants on protection from changes in kidney function (eGFR), but the increase in osteopontin at Day 3 post cisplatin may reflect subacute injury prior to the later improvement in eGFR.

The transporters MATE1 and MRP2 are brush border proteins that efflux cisplatin from proximal tubule cells to urine and reduce the susceptibility to nephrotoxicity [18,19]. A previous publication noted that the *SLC47A1* rs2289669 variant was linked to hematological toxicity secondary to platinum containing chemotherapy [37]. Using oxaliplatin as a substrate, an in vitro study with *SLC47A1*-transfected variants in HEK-293 cells demonstrated loss of MATE1 function [42]. Several publications have reported on variants in *SLC47A1* and the pharmacokinetics and pharmacodynamics of metformin [43,44,45,46]. The presence of the *SLC47A1* polymorphism rs2289669 resulted in higher area under the plasma concentration time curve and lower renal clearance of metformin [44]. In the current study, the *SLC47A1* rs2289669 variant was correlated with increases in the urinary biomarkers KIM-1 and MCP-1. Decreased function polymorphisms in *SLC47A1* are important for potentially increasing therapeutic efficacy, reducing renal clearance, and enhancing kidney toxicity of pharmaceutical substrates of MATE1.

Polymorphisms in the *ABCC2* gene have been shown to affect medication efficacy and safety. Variants in the *ABCC2* gene have been purported to influence the therapeutic efficacy of anti-epileptic drugs [47,48,49,50]. Other studies have identified an association between variants in the *ABCC2* gene and tenofovir induced kidney tubular dysfunction [51]. The *ABCC2* rs717620 variant has previously been linked with responses to platinum chemotherapy [37]. However, in cancer patients, one study failed to demonstrate a relationship between polymorphisms in *ABCC2* and cisplatin pharmacokinetics, which may be due to low statistical power or the use of traditional AKI endpoints [52]. In the current study, several *ABCC2* variants were correlated with increased urinary biomarkers of AKI in patients receiving cisplatin; however, no relationships were observed with eGFR.

Significantly greater increases in urinary calbindin, clusterin, cystatin C, and NGAL at Day 3, and calbindin at Day 10 were observed in *ABCC2* variant rs3740066. Patients heterozygous for the *ABCC2* variant rs717620 exhibited increases in clusterin (3.5-fold) and cystatin C (2.6-fold) at Day 3. The current study in patients receiving cisplatin chemotherapy and published data from patients with epilepsy and HIV-1 support the influence of polymorphisms in *ABCC2* on biomarker changes and outcomes such as efficacy and toxicity.

Glutathione S-transferases metabolize platinum chemotherapeutics including cisplatin. The *GSTP1* Ile105Val polymorphism was previously purported to be associated with less neuropathy [53] and clinical outcomes [54] related to oxaliplatin. The current study reported associations in the same *GSTP1* variant (rs1695) and significant increases in the urinary biomarkers KIM-1, Calbindin, and NGAL at Day 3 and IL-18 at Day 10 after cisplatin chemotherapy. While polymorphisms in GST metabolism genes appear to have some significance with biomarker changes, further study is required to fully evaluate the influence of metabolism in the presence or absence of transporter haplotypes.

It is known that regulatory genes such as *NFE2L2* and *KEAP1* can influence the expression and function of transporters and drug metabolizing enzymes [34,35]. A previous publication reported a relationship between a promoter variant in *NFE2L2* and susceptibility to ototoxicity with high cumulative doses of cisplatin [55]. The current study found links between *NFE2L2* polymorphisms and the enhanced excretion of urinary AKI biomarkers. The *NFE2L2* polymorphism rs2886162 was related to increases in calbindin at Day 10 vs. homozygous wildtypes. Additionally, homozygous variants of *NFE2L2* polymorphisms rs1806649, rs1962142, rs2886162, and rs2706110 were correlated with statistically significant fold-change increases in IL-18, TFF3, MCP-1, and cystatin C. Because *KEAP1* is related to NRF2, polymorphisms in *KEAP1* were also evaluated for their relationships with urinary biomarkers. TFF3 and cystatin C levels were statistically increased on Day 3 and Day 10 vs. homozygous wildtype patients for *KEAP1* (rs11085735). The interaction between polymorphisms involved in cisplatin regulation, metabolism and transport has the potential to clarify mechanisms of cisplatin induced kidney injury.

The strength of the study lies in the comprehensive and targeted genomic approach to evaluating cisplatin induced kidney injury based on the hypotheses that genomics of drug transport and metabolism are a central component of this toxicity. This study incorporated polymorphisms involved in both processes as well as in regulation of these processes. Additionally, the current study evaluated kidney injury using both traditional measures and a panel of novel urinary biomarkers. However, several limitations exist for this study. This study was primarily comprised of Caucasian patients, which reduces its generalizability to other races. Due to the limited number of non-Caucasian races, genotype frequency data (Table 2) were assessed based on the published data for Caucasians. The patients in the study received low to moderate doses of cisplatin, potentially limiting our ability to detect clinical nephrotoxicity (as evidenced by elevations in serum creatinine) and associations between urinary biomarkers and pharmacogenetic variants. While the current study assessed relationships between genomics of selected drug metabolism and transport genes important for the disposition of cisplatin, the role of these polymorphisms in other forms of drug induced kidney injury may not be directly applicable and requires further study. This study was also not designed to study cancer specific outcomes such as progression free or overall survival based on genotype. 

## 4. Materials and Methods 

### 4.1. General Reagents

FlexiGene^®^ DNA Kits (ID# 51206) for DNA extraction from blood were purchased from QIAGEN Inc. (Germantown, MD, USA). Calbindin, clusterin, KIM-1, GST-P1, IL-18, MCP-1, albumin, B2M, cystatin C, NGAL, osteopontin, and TFF3 assays (Bio-Plex Pro RBM human kidney toxicity assay panels 1 and 2) were purchased from Bio-Rad, Life Science (Hercules, CA, USA).

### 4.2. Study Population

Eligible patients were greater than 18 years old and were treated with intravenous cisplatin for treatment of solid tumors in outpatient clinics at the University of Colorado Cancer Center, Aurora, CO, a National Cancer Institute–Designated Consortium Comprehensive Cancer Center. Patients received intravenous cisplatin in doses of ≥20 mg/m^2^. Other inclusion criteria were hemoglobin ≥10 g/dL, no consumption of grapefruit juice or alcohol within 7 days, no history of alcohol consumption of >14 drinks/week, no history of organ transplantation or kidney dialysis, willingness to comply with study, not pregnant or lactating, no changes in medications within previous 4 weeks, and normal liver function (ALT and AST <2 times ULN). As part of the standard of care protocol, patients were hydrated with 0.9% sodium chloride (1–2 L) pre- and post-cisplatin infusion. Exclusion criteria included a diagnosis of kidney cancer, previous exposure to platinum-based chemotherapy, herbal supplement use, exposure to other known nephrotoxins (including contrast agents) within previous 30 days, and concurrent use of inhibitors of cisplatin transport proteins.

Blood was collected for DNA isolation from patients who were either scheduled to receive cisplatin prospectively or had historically received cisplatin treatment. Patient demographics (e.g., race, age, gender, weight, body surface area), pre-chemotherapy laboratory tests (e.g., serum creatinine, estimated glomerular filtration rate [33], blood urea nitrogen, urinary albumin excretion, electrolytes, liver function tests, and complete blood count), medical and medication history, and physical examination data were collected. 

The Institutional Review Boards at the University of Colorado (COMIRB Protocol 12-1510) and Rutgers University (Protocol E13-716) approved protocols for recruitment and sample collection. The investigations were carried out in accordance with the rules of the Declaration of Helsinki [56]. 

### 4.3. DNA Isolation

Blood (5 mL) was collected in heparinized tubes from recruited patients who were scheduled to receive or had historically received cisplatin therapy. Whole blood underwent centrifugation at 2500× *g* for 10 minutes at room temperature to obtain buffy coats for DNA extraction. Resultant buffy coats were aliquoted into 1.5 mL tubes and frozen at −80 °C until DNA isolation. DNA from buffy coats was extracted and purified per FlexiGene^®^ DNA Handbook (QIAGEN) protocol and stored in a stock concentration of 20 ng/mL. DNA was stored at −80°C until subsequent genotyping.

### 4.4. Genotyping

Genotyping was performed using QuantStudio 12K Flex Real-Time PCR System at the University of Utah Genomics Core Facility. Custom Open Array Chips (Thermo Fisher Scientific, Waltham, MA, USA) were designed with selected polymorphisms of interest including transporters (e.g., *SLC22A2*, *ABCC2*, *SLC47A1*), regulatory (e.g., *NFE2L2* and *KEAP1*) and metabolism (e.g., *GSTA1*, *GSTP1*, and *GGT1*) genes. For the Chip, DNA primer sequences were created based on polymorphism ID from NCBI polymorphism Database. Taqman Genotype Software was used to code each genotype as 0 for homozygous wildtypes (wildtype/wildtype), 1 for heterozygous (wildtype/variant), and 2 for homozygous variants (variant/variant) in order to perform statistical analyses. 

### 4.5. Collection of Urine Samples

Urine samples for protein biomarkers were obtained from all patients who were prospectively scheduled to receive cisplatin treatment. Urine samples were collected at baseline (pre-infusion or at 0–2 h after infusion), between 2–5 days (denoted as Day 3) and 9–11 days (designated as Day 10) post-cisplatin infusion. Urine was centrifuged at 3000× *g* and the supernatant was aliquoted for subsequent biomarker assays. All samples were frozen at −80 °C within 30–60 min following collection. At time of analysis, samples were thawed, placed on ice and centrifuged at 1500 rpm for 5 min. Ten µL of supernatant was used for biomarker analyses.

### 4.6. Assessment of Urinary Biomarkers

Urinary samples for protein biomarkers (e.g., calbindin, clusterin, KIM-1, GST-pi, IL-18, MCP-1, albumin, B2M, cystatin C, NGAL, osteopontin, TFF3) were washed using a Bio-Plex Pro II wash station (Bio-Rad) and then analyzed using Bio-Plex, MagPix Multiplex Reader (Bio-Rad). The resultant mean fluorescence intensity (MFI) was used to calculate respective concentrations of each biomarker in the sample. Analysis was completed per protocol with dilutions of 1:10 for panel 1 and 1:50 for panel 2. When concentrations fell outside of the detection limit of the assay, they were diluted and re-analyzed or substituted with the lower limit of quantification divided by 2. The eGFR was obtained from the medical record at baseline and after the first cisplatin dose and calculated using the CKD-EPI equation [33]. Additional details on biomarker analysis methods was previously published [57]. Biomarker data was not normalized to urinary creatinine as our previous study demonstrated similar findings with absolute and normalized data [57]. Biomarker data was assayed at baseline, and Day 3 and Day 10 post cisplatin administration.

### 4.7. Statistical Analysis 

Patient demographic data includes the group mean ± standard deviation. Hardy Weinberg equilibrium was used to analyze allelic frequencies by Chi Square Tests. Differences in eGFR or biomarker changes by genotype groups were evaluated by Students T-test or ANOVA with Dunn’s multiple comparisons post hoc test. Differences were considered statistically significant at *p* < 0.05. Due to the pilot and hypothesis generating nature of the research and the limited sample of patients who had matched genomics and urinary biomarker data, we did not apply any FDR adjustments on *p* values. Patients with missing values were excluded from analysis. All statistical analyses and graphs were completed by GraphPad Prism V6 (GraphPad Software, La Jolla, CA, USA), Partek Genomics Suite (Partek GS 6.4, St. Louis, CA, USA), and SAS 9.4 (SAS Institute Inc., Cary, NC, USA).

## 5. Conclusions

An improved understanding of the role of pharmacogenetic variants that regulate cisplatin transport and metabolism in renal tubule cells may help to reduce the severity of kidney injury and prevent morbidity in patients receiving treatment with cisplatin. The current study facilitates a greater understanding of the influence a patient’s genotype contributes toward nephrotoxic risk in order to potentially inform about a preemptive screen to reduce the risk of kidney damage. Knowledge concerning which polymorphisms are associated with cisplatin induced nephrotoxicity will help to stratify patients at greatest risks to integrate strategies to prevent AKI and improve treatment outcomes in cancer patients.

## Figures and Tables

**Table 1:** Patient Characteristics (*n* = 206).

Data Presented as Mean ± Standard Deviation	
Age	53 ± 14 years
BSA	1.9 ± 0.3 m^2^
Gender	51% male: 49% female
Weight	80 ± 20 kg
Race	92% White: 8% Other
First Cisplatin Dose	59 ± 25 mg/m^2^
Total Dose	479 ± 219 mg
Baseline Serum Creatinine	0.9 ± 0.2 mg/dL
Baseline eGFR	91 ± 21 mL/min/1.73 m^2^
Cancer Etiologies (number, %)	Genital (54, 26%)
	Head and Neck (41, 20%)
	Melanoma (31, 15%)
	Lung (25, 12%)
	Digestive (21, 10%)
	Urinary (18, 9%)
	Other (16, 8%)

Abbreviations: Body surface area: BSA; Estimated glomerular filtration rate: eGFR.

**Table 2:** Genotype and Allelic Frequencies for Transport, Metabolism, and Regulatory Genes Relevant to Cisplatin Disposition (*n* = 206).

Gene	Variant	Homozygous Wildtype Frequency Observed		Heterozygous Frequency Observed		Homozygous Variant Frequency Observed		Undeter-Mined	Major Allele Frequency Observed (Expected)		Minor Allele Frequency Observed (Expected)	
*SLC22A2* (OCT2)	rs316019 *	C/C	0.679	A/C	0.187	A/A	0.014	0.120	C	0.824 (0.79)	A	0.118 (0.21)
	rs3127573	A/A	0.737	A/G	0.211	G/G	0.010	0.043	A	0.858 (0.88)	G	0.10 (0.12)
	rs2279463	A/A	0.665	A/G	0.201	G/G	0.005	0.014	A	0.869 (0.88)	G	0.097 (0.12)
	rs596881	C/C	0.741	C/T	0.230	T/T	0.010	0.019	C	0.861 (0.89)	T	0.098 (0.11)
*SLC31A1* (CTR1)	rs7851395	A/A	0.306	A/G	0.431	G/G	0.187	0.077	A	0.553 (0.53)	G	0.431 (0.47)
	rs12686377	C/C	0.718	A/C	0.158	A/A	0.038	0.086	C	0.847 (0.92)	A	0.194 (0.08)
*SLC47A1* (MATE1)	rs2289669	G/G	0.278	A/G	0.464	A/A	0.196	0.062	G	0.527 (0.54)	A	0.443 (0.46)
*ABCC2* (MRP2)	rs717620	C/C	0.603	C/T	0.258	T/T	0.043	0.096	C	0.776 (0.81)	T	0.207 (0.19)
	rs2273697 *	G/G	0.531	A/G	0.325	A/A	0.053	0.091	G	0.729 (0.82)	A	0.23 (0.18)
	rs3740066	C/C	0.397	C/T	0.368	T/T	0.144	0.091	C	0.63(0.62)	T	0.379 (0.38)
*GGT1*	rs4820599	A/A	0.464	A/G	0.349	G/G	0.100	0.086	A	0.681 (0.73)	G	0.316 (0.27)
*GSTP1*	rs1695 *	A/A	0.354	A/G	0.402	G/G	0.129	0.033	A	0.63(0.59)	G	0.372 (0.41)
*KEAP1*	rs11085735	C/C	0.746	A/C	0.139	A/A	0.014	0.100	C	0.864 (0.91)	A	0.118 (0.09)
	rs1048290	C/C	0.282	C/G	0.407	G/G	0.129	0.182	C	0.531 (0.68)	G	0.359 (0.32)
*NFE2L2* (NRF2)	rs2886162	A/A	0.239	A/G	0.459	G/G	0.220	0.081	A	0.489 (0.58)	G	0.469 (0.42)
	rs1806649	C/C	0.512	C/T	0.311	T/T	0.053	0.124	C	0.716 (0.77)	T	0.23 (0.23)
	rs1962142	G/G	0.000	A/G	0.670	A/A	0.244	0.086	G	0(0.92)	A	0.494 (0.08)
	rs2706110	C/C	0.560	C/T	0.344	T/T	0.086	0.010	C	0.748 (0.8)	T	0.293 (0.2)
	rs6721961	G/G	0.665	G/T	0.225	T/T	0.024	0.091	G	0.815 (0.8)	T	0.155 (0.2)

* Denotes non-synonymous variant.

**Table 3:** Associations between Variant Genotypes and Fold Change Increases in Urinary Biomarkers of Kidney Injury (*n* = 57) *.

Gene	Variant	Protein Biomarker	Time	Fold Change (WT/WT vs. WT/Var)	*p*-value	Fold Change (WT/WT vs. Var/Var)	*p*-value
*SLC22A2* (OCT2)	rs596881	B2M	Day 3	−2.134	0.039	2.474	0.057
		Osteopontin	Day 3	1.918	0.049	−1.052	0.972
	rs316019	KIM-1	Day 3	1.77 × 10^171^	0.038	N/A	N/A
		KIM-1	Day 10	−1.38 × 10^84^	0.046	N/A	N/A
*SLC31A1* (CTR1)	rs7851395	Osteopontin	Day 3	1.341	0.488	2.509	0.015
*SLC47A1* (MATE1)	rs2289669	KIM-1	Day 3	1.379	0.636	3.605	0.007
		MCP-1	Day 3	1.322	0.629	2.952	0.015
*ABCC2* (MRP2)	rs717620	Clusterin	Day 3	3.534	0.024	−2.981	0.854
		Cystatin C	Day 3	2.627	0.034	1.279	0.910
	rs3740066	Calbindin	Day 3	1.900	0.017	1.068	0.883
		Calbindin	Day 10	2.732	0.023	−1.269	0.822
		Clusterin	Day 3	1.170	0.870	4.384	0.012
		Cystatin C	Day 3	1.601	0.447	3.094	0.038
		NGAL	Day 3	2.110	0.030	1.011	0.986
	rs2273697	KIM-1	Day 3	1.153	0.730	5.966	4.29 × 10^−5^
		KIM-1	Day 10	1.163	0.636	2.808	0.042
		Calbindin	Day 3	1.124	0.687	2.648	0.038
		MCP-1	Day 3	−1.215	0.635	3.500	0.010
*GSTP1*	rs1695	KIM-1	Day 3	1.195	0.732	2.690	0.029
		Calbindin	Day 3	1.369	0.354	2.371	0.012
		IL-18	Day 10	−1.566	0.337	2.287	0.012
		NGAL	Day 3	1.294	0.572	2.569	0.027
*KEAP1*	rs1048290	NGAL	Day 3	−1.849	0.211	2.051	0.035
	rs11085735	Calbindin	Day 10	2.944	0.019	−17.366	0.661
		Cystatin C	Day 3	3.275	0.011	1.207	0.928
		Cystatin C	Day 10	2.600	0.027	−2.481	0.751
		TFF3	Day 3	1.747	0.038	1.466	0.619
		TFF3	Day 10	1.776	0.034	−1.629	0.685
*NFE2L2*	rs2886162	Calbindin	Day 10	3.486	0.029	1.53	0.710
	rs1806649	IL-18	Day 3	−1.298	0.692	3.744	0.002
		TFF3	Day 3	−1.133	0.709	2.337	0.004
	rs1962142	TFF3	Day 10	−1.638	0.028	N/A	N/A
	rs2706110	MCP-1	Day 10	1.236	0.621	3.182	0.002

* Only significant figures included. Abbreviations: β-2 microglobulin: B2M; Glutathione-s-transferase pi 1: *GSTP1*; Interleukin 18: IL-18; Kidney Injury Molecule 1: KIM-1; Monocyte chemotactic protein 1:MCP-1; Neutrophil gelatinase-associated lipocalin: NGAL; Trefoil factor 3: TFF3; Variant: VAR; Wildtype: WT.
